# Fibroma of Tendon Sheath Presenting Limited Flexion of the Fingers

**DOI:** 10.1155/2017/4129714

**Published:** 2017-05-16

**Authors:** Kazuhiro Maeda, Nokitaka Setsu, Yoshiharu Kato, Akira Kawai, Eisuke Kobayashi

**Affiliations:** ^1^Division of Musculoskeletal Oncology, National Cancer Center Hospital, Tokyo, Japan; ^2^Department of Orthopaedic Surgery, Tokyo Women's Medical University, Tokyo, Japan

## Abstract

A 35-year-old Japanese man presented with a 1-month history of limited flexion and radiating pain in the left middle and ring fingers. A physical examination revealed a hard nodular mass in his left palm. Magnetic resonance imaging showed a 2 × 1.5 × 1 cm mass of low intensity on T1-weighted images and high intensity on T2-weighted images and gadolinium enhancement. The tumor was marginally resected, adhering to the flexor digitorum profundus of both the third and fourth fingers. The histological diagnosis was fibroma of tendon sheath. After the surgery, the range of motion and hand function were improved. No recurrence has been observed. Fibroma of tendon sheath usually arises on the fingers and hands with strong attachment to the tendon or tendon sheath. The tumor in the present case probably limited the range of flexion of the fingers by obstruction of the transverse carpal ligament.

## 1. Introduction

Fibroma of tendon sheath is an uncommon soft tissue tumor presenting as a solitary, slow-growing, firm, painless, small nodule that shows strong attachment to the tendon or tendon sheath [1–4]. It is usually localized on the finger and hand tendons in adults between 20 and 50 years of age [2]. 98% of these tumors originate in the extremities, with 81.8% of those in the extremities occurring in the fingers, hands, or wrists. The tumors were more likely to be located on the flexor surface of the hand and to be located in the right hand [2]. Surgery for local excision should be performed carefully, as the recurrence rate is 24%, and all of the cases are in the hands and fingers [5].

About one-third of the cases present with tenderness and mild pain due to compression of the nerves underlying fibroma of tendon sheath [2]. Fibroma of tendon sheath has also been reported to cause a “trigger wrist” [6–8], an impingement of the tumor adhering to the flexor tendons in the carpal canal, resulting in snapping finger or carpal tunnel syndrome. However, there have been few reports of the tumor causing complete flexion limitation of the finger. We herein present a case of fibroma of tendon sheath provoking limited flexion of multiple fingers.

## 2. Case Report

A 35-year-old right-handed Japanese man presented with a 1-month history of limited flexion and radiated pain in the left middle and ring fingers. He discontinued his work as a dentist because of the symptoms. A physical examination demonstrated an elastic hard nodular mass in his left palm with radiating pain. The active motion of the middle and ring fingers revealed loss of full flexion. The finger-to-palm distance was 10 mm. The passive motion was not restricted in all of the joints of the fingers. The left hand function was also reduced, with a grip strength of 35 kg in the right hand and 5 kg in the left. The Disabilities of the Arm, Shoulder and Hand (DASH) score was 14.5. The box and blocks test (BBT) score was 75 in the right hand and 55 in the left.

**Figure 1:**
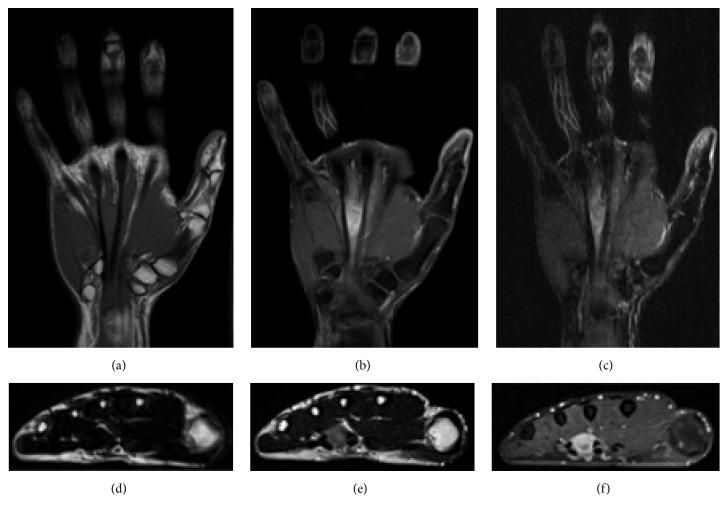
Magnetic resonance imaging revealed a 20 × 15 × 10 mm mass of low intensity on T1-weighted images (a, d) and high intensity on T2-weighted images (b, e) and gadolinium enhancement (c, f).

The laboratory test findings, including blood cell count and blood chemistry, were all within normal ranges. Radiographs of the hand revealed no remarkable findings. Magnetic resonance imaging (MRI) revealed a 2 × 1.5 × 1 cm mass of low intensity on T1-weighted images and high intensity compared with normal muscle on T2-weighted images and gadolinium enhancement (Figure 1). 2-[^18^F]-Fluoro-2 deoxy-D glucose positron emission tomography revealed increased uptake with a maximum standardized uptake value of 3.73 in the mass.

**Figure 2:**
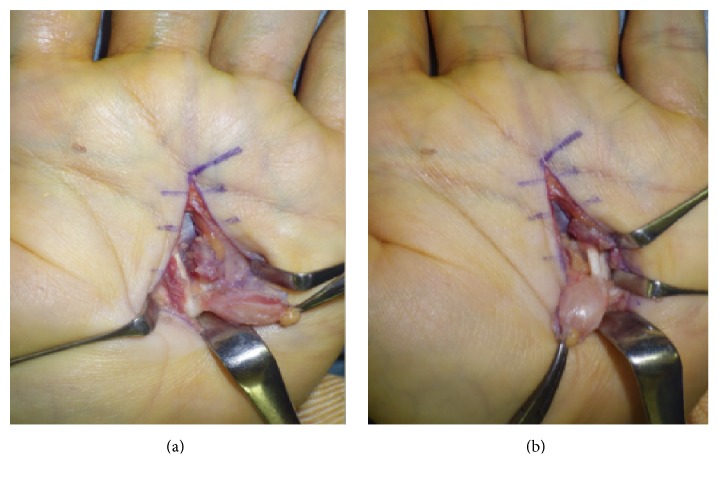
(a, b) The tumor adhered to the flexor digitorum profundus of both the third and fourth fingers.

After biopsy, he underwent resection of the tumor. The tumor had adhered to the flexor digitorum profundus of both the third and fourth fingers (Figure 2). From which tendon the tumor arose could not be determined from the operative findings. The tumor, which was grossly firm and white-colored, was carefully dissected from the flexor tendons and neurovascular structures.

**Figure 3:**
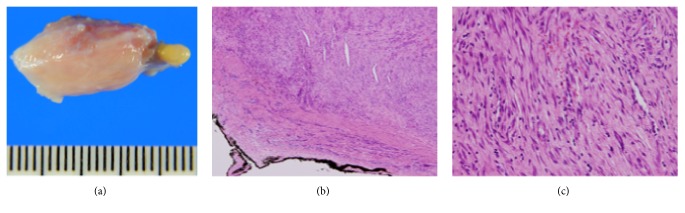
(a) The external surface of the mass was smooth and glistening without any gross abnormalities or disruption. (b, c) The lesion was well circumscribed and contained fibroblastic spindle cells. Elongated, slit-like spaces were prominent (hematoxylin-eosin, original magnification 20x, 400x).

The external surface of the mass was smooth and glistening without any gross abnormalities or disruption and measured 2.0 × 1.3 × 0.9 cm (Figure 3). The lesion was well circumscribed and contained fibroblastic spindle cells in a collagenous background, and some inflammatory cells were seen. There was generally no atypia. Elongated, slit-like spaces were prominent (Figure 3). *β*-Catenin immunohistochemistry showed no nuclear staining. The histological diagnosis was fibroma of tendon sheath.

Immediately after the surgery, the limited flexion of the fingers was improved. The patient achieved full range of motion of all of the fingers with no pain or tenderness by two months after the surgery. The left hand function was also improved, with a postsurgery grip strength of 24 kg. His DASH score was 0.75, and his BBT score was 73 in the left hand. His International Society of Limb Salvage (ISOLS) score was 30. He returned to his dental practice, and no recurrence has been observed in the two years since the surgery.

## 3. Discussion

Certain tumors more likely to be located in the soft tissue in the hand than in other parts of the body include glomus tumor, tenosynovial giant cell tumor, and fibroma of the tendon sheath [9]. However, fibroma of the tendon sheath is uncommon among these tumors [4, 9].

The differential diagnosis of fibroma of tendon sheath may include epidermal cyst, mucinous cyst, neuroma, leiomyoma, nodular fasciitis, synovial sarcoma, and tenosynovial giant cell tumor. The clinical features of tenosynovial giant cell tumor are particularly similar to those of fibroma of tendon sheath. Both are likely to occur in the fingers with attachment to tendon sheath, possess similar MRI signals, and have firm, well-circumscribed, multilobulated gray-white appearances [4, 10]. However, fibroma of tendon sheath is distinguished from tenosynovial giant cell tumor by its histopathologic features, which include the fact that giant cell tumors of tendon sheath are less hyalinized and more cellular than fibroma of tendon sheath and have histiocytes and monocytes as well as multinucleated giant cells, foam cells, and hemosiderin-laden macrophages.

Fibroma of tendon sheath shows low signal intensity in both T1- and T2-weighted MRI images when in contact with the tendon sheath [10]. However, the T2-weighted findings are not constant and can have mixing of low and high intensity signals, uniformly low intensity signals, and a high intensity signal with low intensity near the edge. This diversity in the signal intensity is considered to reflect the varying density of the collagen fibers.

The gross findings of fibroma of tendon sheath are that the lesion is well circumscribed, nodular to multinodular, usually less than 2 cm in diameter, and composed of firm gray-tan tissue [2]. The microscopic findings are a lobulated form containing bland fibroblastic spindle cells in a collagenous background [2]. There is generally no atypia. The cellularity is usually low but can be variable and is often higher at the edge, sometimes resembling nodular fasciitis. There are characteristic elongated (slit-like) thin-walled vessels or clefts. Degenerative features such as myxoid changes, chondroid or osseous metaplasia, or bizarre pleomorphic cells can be observed.

Soft tissue tumors around the hands have been reported to evoke a functional disorder known as “trigger wrist.” “Trigger wrist” is a very rare condition generally characterized by a painful click or catching sensation around the wrist joint during finger or wrist motion [8]. It is caused by pathology under or around the flexor retinaculum and has several etiologies, including tumors, tenosynovitis, and anomalous muscle bellies [7, 8]. The tumors known to cause “trigger wrist” are fibroma of tendon sheath and tenosynovial giant cell tumor, both of which tend to show adherence to the tendon [8]. While a few cases of these tumors presenting with “trigger wrists” have been published [7, 8], to our knowledge, none have shown limited flexion.

The fibroma of tendon sheath in the present case, which adhered to the deep flexor tendons of both the third and fourth fingers, existed at the distal portion of the transverse carpal ligament. When the patient tried to flex these fingers, the tumor was likely caught by the distal edge of the transverse carpal ligament, preventing the patient from flexing the fingers completely. The tumor was probably located so distally that it could not cause “trigger wrist” at first, but the location between two tendons allowed the tumor to adhere to both tendons and increased the “mass effect,” so that the tendons and the tumor became stuck in the distal end of the carpal tunnel. Surgical excision of the tumor eventually made complete finger flexion possible.

We herein presented a case of fibroma of tendon sheath presenting with limited flexion of the third and fourth fingers. A neoplastic lesion should thus be considered in the clinical differential diagnosis of limited range of motion of the fingers.
